# What changes in the biology of bone movement induced with mini-implants/miniplates is the synchronicity

**DOI:** 10.1590/2177-6709.27.3.e22ins3

**Published:** 2022-07-04

**Authors:** Alberto CONSOLARO, Ertty SILVA, Maurício de Almeida CARDOSO

**Affiliations:** 1 Universidade de São Paulo, Faculdade de Odontologia de Bauru (Bauru/SP, Brazil).; 2 Universidade de São Paulo, Faculdade de Odontologia de Ribeirão Preto, Programa de Pós-graduação em Odontopediatria (Ribeirão Preto/SP, Brazil).; 3 Faculdade de Medicina e Odontologia São Leopoldo Mandic, Programa de Pós-graduação em Ortodontia (Campinas/SP, Brazil).

**Keywords:** Induced tooth-bone movement, Induced tooth movement, Orthodontic movement, Osteocytes, Osteocytic network

## Abstract

**Introduction:**

Induced tooth-bone movement occurs by a synchronicity of dental and bone phenomena, thanks to the osteocytic network, which is a three-dimensional network that controls the bone shape or design.

**Objective:**

To describe the tooth-bone movement induced by enhanced anchorage, divided into three distinct moments: zero, start and stop.

**Question:**

From this description, the main question arises: with the use of mini-implants/miniplates, what changes in the biology of induced tooth-bone movement? The answer is: nothing changes, either biologically or microscopically.

**Conclusion:**

This technique optimizes the treatment time, and the range of therapeutic possibilities is broadened, thanks to the synchronicity of phenomena - which remain the same, in all teeth and bones, yet in a synchronized manner. Bone anchorage represents synchronicity in induced tooth-bone movement.

## RESUMO

**Introdução:**

A movimentação osseodentária induzida ocorre meio de uma sincronicidade de fenômenos dentários e ósseos, graças à rede osteocítica, uma rede tridimensional de controle do formato ou *design* ósseo.

**Objetivo:**

Descrever a movimentação osseodentária induzida com ancoragem ampliada, dividindo-a em três momentos distintos: zero, *start* e *stop*.

**Questionamento:**

Dessa descrição origina-se a principal pergunta: com o uso de mini-implantes/miniplacas, o que muda na biologia da movimentação osseodentária induzida? A resposta é: não muda nada, nem biologicamente, nem microscopicamente.

**Conclusão:**

O que se otimiza, com essa técnica, é o tempo de tratamento, e se amplia o leque de possibilidades terapêuticas, graças à sincronicidade dos fenômenos - que continuam sendo os mesmos, em todos os dentes e nos ossos, só que de forma sincronizada. A ancoragem óssea representa a sincronicidade na movimentação osseodentária induzida.

## INTRODUCTION

For decades, induced tooth-bone movement was called induced “tooth” movement; however, considering the current knowledge, it is known that it involves synchronized bone and tooth phenomena. Teeth change their position thanks to changes in bone volume and shape.

Many doubts arise when trying to understand the induced tooth-bone movement in different clinical situations, such as in conventional orthodontic treatment with brackets and wires, and in orthodontic treatment with the use of mini-implants and miniplates to obtain enhanced anchorage.

In both situations, the molecular, cellular and tissue phenomena are the same, including intensity and duration. The difference is that, in enhanced anchorage, there is synchronicity in the most varied points of the teeth and bones, i.e., the phenomena occur at the same time in several sites.

## APPLIED FORCES: DISTRIBUTION X INTENSITY

One concern of clinicians is that enhanced anchorage appliances use heavier forces than conventional treatments. For tissues and cells, what matters are the forces that reach them directly.^1^ ^-^ ^4^

At the moment of activation of enhanced anchorage appliances, the applied load is greater than the conventional load, yet it is distributed along the teeth and bones, being reduced when reaching the tissues.

What matters the most is not the intensity, but the distribution of forces acting on the tissues. Unfortunately, there is no way to measure the intensity of forces directly on the cells and tissues - we have not yet reached this technological advance -, but rather those applied at the moment of activation of appliances, wires and elastics.

In induced tooth-bone movement, the frequency and intensity of iatrogenic tooth resorption are more associated with the concentration and/or distribution of forces than with their intensity.^5^ ^,^ ^6^ ^,^ ^7^ Intense well-distributed forces are better accepted and cause less tissue damage, while lower forces concentrated in a smaller area tend to cause more damage.

In the induced tooth-bone movement with enhanced anchorage, the applied force is distributed more uniformly across the bone and tooth structures, justifying why tooth resorptions are less frequent and intense in orthodontic treatments with miniplates than those observed in conventional treatments.^8^ ^-^ ^12^

## THE THREE MOMENTS OF INDUCED TOOTH-BONE MOVEMENT USING ENHANCED ANCHORAGE

To understand the synchronicity of cellular and tissue phenomena in induced tooth-bone movement, we established three moments for their characterization, namely:

### ZERO MOMENT: THE NORMAL STRUCTURES

Nearly 50% of the periodontal ligament volume is occupied by microcirculation vessels. In its 0.25-mm thickness, it is laterally profiled by cementoblasts on the root surface, and by osteoblasts and medullary spaces on the surface of alveolar bone that lines the alveolus internally and continues, without limits, with the secondary bone of the alveolar process.

The collagen fibers of the ligament are roughly parallel to each other, interspersed by the fibroblasts that produced them. Among the fibers there are also other cell types, such as tissue stem cells and occasional leukocytes.

Very slowly, cementoblasts produce very thin layers of cementum matrix daily, renewing the collagen fibers that are inserted into it. On the other side of the ligament, osteoblasts also deposit bone matrix of alveolar bone, also to continuously insert periodontal collagen fibers, which are renewed every day. This process of constant renewal of periodontal and bone structures justifies why 50% of the ligament volume is composed of vessels.

The alveolar bone proper, also known as primary or embryonic bone, remains present only in the periodontal ligament and tendon insertions. Its renewal rate is more accelerated, and its structure with abundant cells forms thin layers juxtaposed to the surface that covers the alveolus, providing dynamism to the insertion and reinsertion of periodontal collagen fibers, meeting the functional demands.

In tissue sections of fasciculated alveolar bone, it is common to observe bone remodeling units, with clasts actively present and participating in the constant process of bone remodeling.

With no defined limits, the alveolar bone continues with the secondary, or mature, bone of the alveolar process, and together they form the alveolar cortical bone, which, in radiographic and tomographic images, is called lamina dura. Bone trabeculae arise from the lamina dura that delimit the medullary spaces filled with fibrous, fatty and/or hematopoietic connective tissue. These spaces are lined by a thin layer of tissue with abundant cells called endosteum.

The periosteum is fundamental in induced tooth-bone movement. In the cervical portions of the alveolus, at the alveolar crests, the alveolar cortical bone is continuous with the outer cortical bone, which is covered externally by fibrocellular tissue called periosteum. Besides being richly vascularized, the periosteum has a very fibrous outer layer, organized into a capsule, to protect the bone externally. Internally, the periosteum adheres to the cortical bone that it produces, by the insertion of many fibers, among which there are many osteoblasts and osteoclasts.

The surface of all bones is covered by periosteum, which protects them and assigns them a high reactive and productive capacity. The periosteum is “absent” in only two situations: in the direct insertions of tendons and in the alveolar bone surface of teeth. In the alveolar bone that lines the tooth alveolus, the periodontal ligament represents a highly specialized periosteum and plays its role in dentoalveolar physiology.

**Figure 1:**
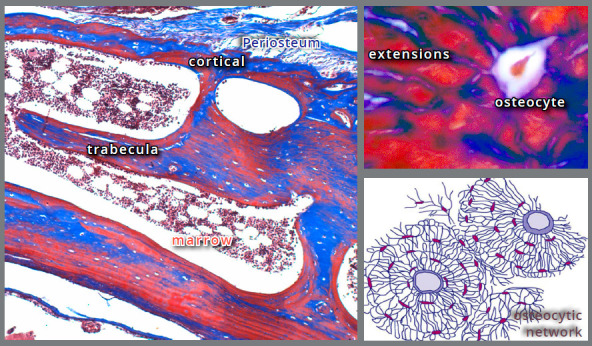
Osteocytes communicate with up to 50 other cells, with their extensions, forming a three-dimensional network that controls bone design according to the induced functional demand, from the phenomena of bone deposition and new formation (Mallory staining: 25 and 100X).

All bone structures have an osteocytic network.^13^ In all aforementioned bone components, inside their structures there are many embedded cells, shaped as spiders, with 20 to 50 cytoplasmic processes, which contact another 20 to 50 cells. These cells are the osteocytes, and they form a three-dimensional intercommunication network to control and change the bone shape or design, based on functional demands received throughout life (Fig 1).

The osteocytic network captures changes in shape and forces applied to any part of bone. The compression and tension forces applied to the bone are captured regardless of their origin, and the bone structure tends to adapt and readjust by the action of mediators and cell-cell messages released by osteocytes.

The osteocytic network communicates, via cytoplasmic processes and mediators, with osteoblasts on the cortical and trabecular surfaces, controlling the deposition of new layers of bone matrix or bone resorption on their surfaces.

Bone remodeling must be constant to keep the bone level of calcium, phosphate and other ions within normal limits. The source of these minerals is food or bone tissue, which explains the constant bone formation-resorption. This feature allows using this phenomenon of bone remodeling to reshape the bone according to functional and esthetic needs, directing and applying forces for this purpose.

Roots are not part of bone remodeling.^6^ Teeth have cementoblasts and cementocytes in their root structure, yet these cells do not make connections with the osteocytic network, not entering the process of constant remodeling as occurs in bone, nor using the teeth as mineral reservoirs for the organism. The osteocytic network is distant from the teeth and on the other side of the periodontal ligament, and it cannot act on dental cells, not even by a distant mechanism.

Likewise, cementoblasts and cementocytes do not have membrane receptors for bone remodeling mediators released by osteocytes and other bone cells, and cannot obtain information or “orders” from the osteocytic network to promote tooth remodeling, as occurs in bones.

When there is alveolodental ankylosis, the replacement resorption following it represents exactly the bone remodeling occurring in dental tissues, since these protective mechanisms or characteristics have been disrupted.

### START MOMENT: THE DYNAMICS OF MOVEMENT HAPPENING

**Figure 2:**
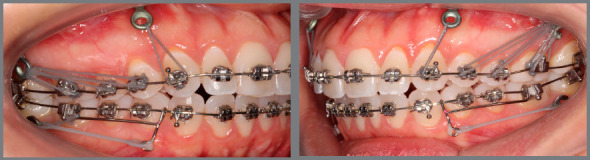
The use of miniplates offers enhanced anchorage and allows uniformly distributing forces in dental and bone tissues, to promote planned remodeling in the shape of the maxilla and mandible, including the dental arches, as observed in this patient.

The activation of forces promotes a deformation of cells and bone components, which will be detected by the three-dimensional osteocytic network, which is modified and deformed by the induced change in position (Figs 1 and 2). By the deformation caused, this osteocytic network knows where to deposit or resorb bone, to adapt to this new functional demand, natural or induced.

In the periodontal ligament, the induced tooth-bone movement promotes compression or stretching of collagen fibers and other ligament structures. The constant shape of cells, maintained by their protein cytoskeleton, is inevitably deformed, and this induces cellular stress, with large release of mediators.

In this state of stress, the amount of cellular intercommunication mediators increases markedly, including the mediators that induce bone resorption and new formation, increasing the rate of bone remodeling in periodontal tissues. These mediators include prostaglandins, cytokines and growth factors, which act only locally and not systemically, since their molecular lifetime is very short. That is why induced tooth-bone movement has no systemic implications, signs and symptoms.

In almost all current cases of induced tooth-bone movement, there is not an established inflammatory process; the exudate and inflammatory infiltrate that can be identified microscopically and characterized as such do not form. Currently, the possibility of extensive hyaline areas and distant bone resorptions, with severe dental resorptions, is lower, thanks to the built-in technology and the manner through which the high-tech orthodontic appliances available are used.

In fasciculated and secondary alveolar bone, the increased amount of mediators accelerates bone remodeling so that the tooth will change its position gradually, by organizational change of the surrounding bone, as well as by the force changing the tooth position in the alveolus. This targeted bone remodeling uses teeth as a lever to transmit the forces that lead to induced tooth-bone movement. With each movement by the action of forces, the dental and bone spatial position is changed in a synchronized manner.

At the same time, with admirable synchronicity, bone deposition and resorption will also occur in the trabeculae and cortical bones distant from the site of force application, represented by the teeth. The osteocytic network, by capturing the effected changes, redesigns the bone trabeculae, communicating throughout the involved bone.

For example, the alveolar cortical bone waits for the tooth to arrive with new layers deposited by the periosteum that was stimulated by the osteocytic network, even at distance, by the cell-to-cell contact of surrounding osteocytes and/or by the mediators released. At the same time, the trabeculae redesign their distribution to better dissipate forces and meet the captured functional demand (Fig 1).

From time to time, after 40 to 60 days of activation in bone anchorage, spatial changes in bone position, volume and shape, as well as in the tooth-bone and tooth-tooth relationships, are observed (Fig 2).

### STOP MOMENT: IT’S TIME FOR NEW TENSEGRITY

When the application of forces in enhanced anchorage is interrupted, the accelerated bone remodeling returns to its normal rate. With a new design or shape, the bones and dental arch will now be in a new tensegrity, which is expected to have been established in the jaws with the facial bones and in the jaws with teeth in their functions. The enhanced anchorage allows a greater diversity of movements and anchorages, expanding the horizon of possibilities.

Tensegrity is the balance between all forces generated in a system or objects, whose resultant is equal to zero, soon after functions and movements are performed. If a new tensegrity in the jaws is not obtained after orthodontic and/or orthopedic treatment, either conventional or with enhanced anchorage, there is a tendency for the system to provide movements and readaptations to return to the previous tensegrity, and thus relapses and/or instabilities occur.^1^ ^-^ ^4^

In orthodontic planning, it is essential to program a new tensegrity, considering all factors that generate movements and tensions, such as the TMJs, occlusion, adjacent teeth, gingiva, soft tissues; and functions such as chewing, swallowing and speaking. With enhanced anchorage, this can be achieved in a shorter treatment time, yet the biology of phenomena involved in induced tooth-bone movement is the same. The important thing is synchronicity and the goals to be achieved.

When enhanced anchorage is applied, the force is distributed over a larger area, and the distribution of this force and its dissipation tends to be more uniform and homogeneous. For the tissues, the forces are similar or even more uniform than those obtained by conventional appliances.

## FINAL CONSIDERATIONS

### SYNCHRONICITY IS NOT SIMULTANEITY

During enhanced anchorage, the osteocytic network allows the force-stimulated bone a synchronicity that cannot be called simply simultaneity^13^ (Fig 1).

Simultaneity suggests independent phenomena occurring at the same time, without necessarily indicating a relationship of goals and meanings between them, or a common cause. Simultaneity is almost the expression of what happens “by chance”.

Conversely, synchronicity indicates the occurrence of phenomena at the same time but maintaining an interrelation of meanings and objectives with each other and with the same cause. In the case of enhanced anchorage with miniplates/mini-implants, the cause is the forces planned and generated with the same objectives.

In argumentation about orthodontic treatments with or without mini-implants/miniplates, questions arise such as:

*- With the use of mini-implants and miniplates, what changes in the biology of induced tooth-bone movement?*

The answer is:

*- Nothing changes, biologically or microscopically. This technique optimizes the treatment time, and the range of therapeutic possibilities is expanded, thanks to the synchronicity of phenomena.*

In conventional orthodontics, movements are limited to few teeth in each treatment phase, and the results occur almost tooth by tooth, taking a longer time. The enhanced anchorage allows the phenomena to occur in a synchronized manner, at the same time and with common goals. Thus, the treatment time decreases, but biologically the phenomena remain the same in all teeth and bones, yet they occur in a synchronized manner. Bone anchorage represents synchronicity in induced tooth-bone movement.
